# Drawing on the Past to Shape the Future of Synthetic Yeast Research

**DOI:** 10.3390/ijms21197156

**Published:** 2020-09-28

**Authors:** Thomas A. Dixon, Isak S. Pretorius

**Affiliations:** 1 Department of Modern History, Politics and International Relations, Macquarie University, Sydney, NSW 2109, Australia; thom.dixon@mq.edu.au; 2 Chancellery and ARC Centre of Excellence in Synthetic Biology, Macquarie University, Sydney, NSW 2109, Australia

**Keywords:** biodesign, biodiversity, biofoundry, consilience, engineering biology, fermentation, scientific method, synthetic genomics, timeline, yeast

## Abstract

Some years inspire more hindsight reflection and future-gazing than others. This is even more so in 2020 with its evocation of perfect vision and the landmark ring to it. However, no futurist can reliably predict what the world will look like the next time that a year’s first two digits will match the second two digits—a numerical pattern that only occurs once in a century. As we leap into a new decade, amid uncertainties triggered by unforeseen global events—such as the outbreak of a worldwide pandemic, the accompanying economic hardship, and intensifying geopolitical tensions—it is important to note the blistering pace of 21st century technological developments indicate that while hindsight might be 20/20, foresight is 50/50. The history of science shows us that imaginative ideas, research excellence, and collaborative innovation can, for example, significantly contribute to the economic, cultural, social, and environmental recovery of a post-COVID-19 world. This article reflects on a history of yeast research to indicate the potential that arises from advances in science, and how this can contribute to the ongoing recovery and development of human society. Future breakthroughs in synthetic genomics are likely to unlock new avenues of impactful discoveries and solutions to some of the world’s greatest challenges.

## 1. The Transformative Impact of the Scientific Method and the Concept of Consilience

Serendipity presumably played an important part in prehistoric inventions and innovations. In preliterate societies, ancient knowledge, and technique of nameless inventors of controlled fire, shelter, clothing, irrigation, the sharp blade, and wheel were passed from generation to generation in an oral tradition [1]. Over time, the ancients of the Archaic Era accumulated valuable knowledge for survival but made no distinction between reason and magic. However, with the invention of the first alphabet and development of writing, discoverers and inventors acquired the skill to store and communicate knowledge across generations with much greater accuracy. This accelerated progress in the Ancient Near East and Mediterranean communities [2].

In Classical Antiquity (8th century bce to 6th century ce), the interlocking civilisations of ancient Greece and ancient Rome started to develop a line of inquiry into the workings of the universe aimed at such practical objectives as establishing a reliable calendar or determining how to cure a variety of ailments. Those abstract investigations were known as natural philosophy. Thales of Miletus (624–546 bce), was the first Natural Philosopher to postulate non-supernatural explanations for natural phenomena and hence became known as the Father of Science. Subsequently, the deductive reasoning of natural philosophers, such as Pythagoras (580–500 bce), Socrates (470–399 bce), Hippocrates (460–370 bce), Plato (424–348 bce), Aristotle (384–322 bce), and Archimedes (287–212 bce) [3,4,5,6,7,8] had a profound influence on later scientific inquiries and investigations during the Hellenistic Age—the period of Mediterranean history between the death of Alexander the Great in 323 bce and the rise of the Roman Empire as marked by the Battle of Actium in 31 bce and the conquest of Ptolemaic Egypt the following year [9].

Under the darkness of cultural decay and intellectual erosion that prevailed between the fall of the Roman Empire in 476 ce and the beginning of the Renaissance in the 14th Century—a period known as the Dark Ages or Middle Ages—there were virtually no scientific advances, except for some notable contributions from Islamic mathematicians. Progressing from medieval darkness to the light of the Renaissance, the Age of Enlightenment (1400–1600) marked an era of cultural, political, economic, and scientific ‘rebirth’. This epic reawakening reacquainted intellectual thinkers of the time with classical philosophers of the previous Greco-Roman, Byzantine, and Middle Eastern worlds. Significant technological and scientific feats were also achieved elsewhere in the world. For example, the feat of colonising the Pacific Islands still stands out as one of this era’s most advanced uses of astronomy, observation of the maritime biome, and ship building [2]. 

**Figure 1:**
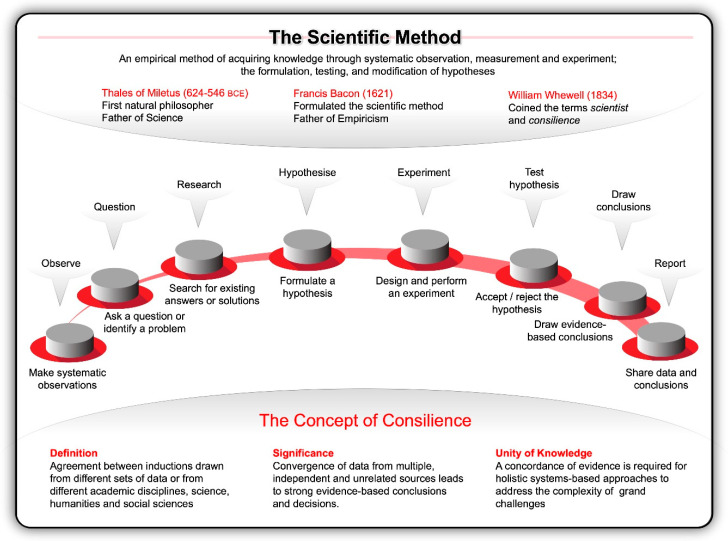
The scientific method, underpinned by the principles of consilience, was perhaps the single most important development in modern history. This approach entails systematic observation, measurement, and experiment, and the formulation, testing, and modification of hypotheses by drawing on data and evidence from independent, unrelated academic disciplines—from science, technology, engineering, mathematical, and medical (STEMM) fields of research to the humanities, arts, and social sciences (HASS).

Inspired by the work of pioneers like Nicolaus Copernicus (1473–1543) and Galileo Galilei (1564–1642) at the dawn of the Renaissance, Francis Bacon formulated the concept of a true scientific method in his Instauratio Magna, which was published in 1621 as the Novum Organum Scientiarum [10]. His rational and methodical approach to inquiry rejected philosophical speculations, venerable opinions, reductive logic, and conjecture about the fundamental nature of the physical world; instead he advocated for inductive reasoning, systematic observation and experimentation [11]. He compared experimenters to ants—they collect and use. He likened logicians to spiders, they make webs out of their own substance. The type of investigator that Bacon valued most are like bees, while pollinating the fields, they gather their material from flowers, digest that material and transform it by the power of their own. This orderly way of collecting measurable, empirical evidence in an experiment related to a hypothesis and generating results aimed to support or contradict a theory (Figure 1), marked the beginning of modern science and earned Bacon the title of Father of Empiricism.

As evidenced by pioneering works, such as those by William Gilbert’s De Magnete [12], William Harvey’s Exercitatio Anatomica de Motu Cordis et Sanguinis in Animalibus [13] and Isaac Newton’s Philosophia Naturalis Principia Mathematica, the scientific method remained influential through the Scientific Revolution (1600–1800) and is widely credited as the single most important development in the history of science [14]. It established a way to validate truth at a time when misinformation was the norm. This enabled investigators to navigate the unknown, leading to, for example, Antonie van Leeuwenhoek’s discovery of microscopic single-celled organisms in 1676; Louise Pasteur’s experimental demonstration in 1859 that fermented beverages result from the action of living yeast transforming glucose into ethanol and carbon dioxide; Gregor Mendel’s postulation of the biological principles of heredity in 1866; and Emil Christian Hansen’s isolation of the first pure yeast culture in 1883 [15,16,17,18,19,20,21].

Since then, scientists honed the ability to distil facts about the universe and life by generating theories, then using experimentation to qualify those theories. With the advantage of 20/20 hindsight and looking back at how far science has come since 1833 when William Whewell coined the terms scientist and consilience (concordance of evidence), one cannot help to be amazed by all that humanity has achieved using these approaches. Looking back, it is clear that all of the transformative breakthroughs that shifted the frontiers were catalysed by the orderliness of the scientific method underpinned by consilience—the principle that evidence from independent, unrelated sources can converge on strong conclusions [22]. In the words of Wilson [22], the synthesis of knowledge from different specialised fields of human endeavour unites the sciences and humanities (Figure 1). This unity of knowledge is a powerful dynamo of scientific and technological advances.

## 2. The Effervescent Spirit of Yeast Fermentation

Yeast researchers were amongst the early adopters and beneficiaries of the scientific method and the concept of consilience. They benefited from cross-disciplinary contributions and collaborations—from the 1676 description of yeast cells by a Dutch textile merchant, Antonie van Leeuwenhoek [23], to the independent proposals by a French physical engineer, Charles Cagniard-Latour [24], a medically-trained German investigator, Theodor Schwann [25], and a German pharmacist, Friedrich Kützing, that these microscopic unicellular organisms nourish themselves by the sugar that they ferment. These independent observations of Cagniard-Latour, Schwann and Kützing in 1837 helped Louis Pasteur to disprove Lazzaro Spallanzanni’s spontaneous generation theory—the formation of living organisms without descent from similar organisms—and Justus von Liebig’s mechanistic theory—that physicochemical forces are responsible for fermentation [26,27]. The 17th century founder of the scientific method, Francis Bacon, would have been proud to witness how, 230 years later, Pasteur, rationally and methodically designed his swan-neck flask experiments that enabled his vitalistic conceptions to triumph over the speculative theory of spontaneous generation and the notion that fermentation was purely due to a chemical process as opposed to a biological one [16].

As Von Liebig and Pasteur were debating the chemical-versus-biological nature of alcoholic fermentation, other scientists, such as the German physiologist, Wilhelm Kühne, and zymologist Edward Büchner [28], entered the arena. In 1877, Kühne invented the term enzyme, which comes from Greek ‘leavened’ or ‘in yeast’, and in 1897, Büchner used cell-free extracts from yeast to prove that ‘zymase’ was the biochemical agent responsible for fermentation. For his work that helped bridging the gap between chemical and biological conceptions, Büchner was awarded the Nobel Prize in 1907 [16]. This laid the groundwork for the foundational work of Gustav Embden, Otto Meyerhof, Jakub Parnas, Gert Cori, Carl Cori, Arthur Harden, Karl Lohman, Carl Neuberg, and Otto Warburg that led to the complete elucidation of the biochemical pathway of glycolysis by 1940 [29,30,31,32,33,34,35,36,37,38]. This was the first fully elucidated metabolic pathway. Unsurprisingly, six Nobel Prizes were associated with the ground-breaking research done by these and other remarkable scientists between the two World Wars [21].

It took almost 100 years to unravel the fermentation pathway but once it was fully understood how the action of living yeast cells make bread dough rise and produce alcohol in beer, wine, and spirits, glycolysis was pivotal to major advances in biochemistry, with immense economic applications. Seven millennia before all this was known, the ancients serendipitously harnessed the boiling, foaming, and bubbling activity (descriptive words from which the term yeast derives) to bake their first spongy bread in Ancient Egypt and brew their first tipple from cereal grains and grapes in the Zagros mountains of Mesopotamia. Neither the ancients nor the scientists who unravelled the science behind the fizzy spirit of yeast would have predicted that, today, the annual global production of commercial yeast strains used in the fermentative food and beverage industries exceeds 1.8 million tons [39]. While hindsight is perfect; foresight is not.

## 3. The Untapped Treasure Trove of Yeast Diversity

The development of techniques for producing pure yeast cultures enabled brewers, winemakers and bakers to distinguish between ‘good’ and ‘bad’ yeast [19,40,41]. This was not only of major importance in standardising yeasts for reliable production of beer, wine and bread, but also for accurate descriptions of new species. The taxonomic classification of yeasts between the invention of the genus and species *Saccharomyces cerevisiae* (a globose or ellipsoid unicellular, multilateral budding, saprotrophic ‘sugar fungus’, 2.8 μm in diameter and 3–25 μm in length) by Julius Meyen [42] in 1838 and Paul Lindner’s description of *Schizosaccharomyces pombe* [43] (a rod-shape, unicellular yeast that measure 3–4 μm in diameter and 7–14 μm in length, and that reproduce vegetatively by fission and not by budding) in 1893 was steady but relatively slow [21]. In 1839, Schwann described yeast characteristics which became known as ascospores [44]. In 1857, Miles Berkeley referred to unicellular fungi carrying ascospores in asci as Ascomycetes [45], and in 1870, Max Reess designated *Saccharomyces* an ascomycete [21]. With Hansen’s isolation of a pure brewer’s yeast culture in the early 1880s [46], yeast taxonomy became a practical proposition. By the end of the nineteenth century reports of approximately 200 species had been published—the identity of about half of these species is known today [21].

In 1904, Hansen published a pivotal paper on the systematics of the *Saccharomycetes*, boosting the development of yeast classification [46]. In the same year, Centraalbureau voor Schimmelcultures (CBS) was established as a culture collection of living fungi, and, in 1908, 50 years after Charles Darwin had published on the Origin of Species, Marie Antoine Guilliermond proposed that ascosporogenous yeasts had evolved from ancestral hyphal fungi [21]. As more and more yeast genera and species were discovered and described in the ensuing years, confusion and controversy were rife, especially around the definition of the concept of ‘species’ and the distinction between sexual (e.g., *Saccharomyces* and *Schizosaccharomyces*) and asexual (e.g., *Brettanomyces*, *Kloeckera*, *Rhodotorula*) yeasts.

In 1928, Guilliermond published a dichotomous key for identifying yeasts [47]. The criteria in this key, which embraced 22 genera, included the appearance of vegetative cells; the presence or absence of ascospores (including their number and shape); and the ability to ferment certain sugars [21]. Based on the early work of Hansen, Lindner, Guilliermond and other early pioneers, Albert Kluyver [48] founded the Dutch school of yeast taxonomy in Delft, the same place where Van Leeuwenhoek observed yeast cells for the first time more than 150 years before. Between 1931 and 1984 a series of foundational monographs by Nellie Stelling-Dekker (1931), Jacomina Lodder (1934), Harmanna Diddens and Lodder (1942), and Lodder and Wihelmina Kreger-van Rij (1984) from this Delft-based school had a major impact on yeast taxonomy until the end of the twentieth century [21]. In 1998, the editorship of the fourth edition of The Yeast: A Taxonomic Study [49] moved from The Netherlands to the United States of America. The editors, Cletus Kurzman and Jack Fell, worked collaboratively with 36 other leading yeast taxonomists to progress this field of research by incorporating contemporary nucleic acid studies and establishing invaluable databases and resources.

The overall goal of yeast taxonomists is to classify yeasts to species level; however, identification of individual subspecies or strains is more the focus of yeast technologists and practitioners. In the words of Barnett [21], the sensible naming of yeasts is vital for research, commerce, and medicine. For both yeast researchers and yeast technologists it is important that (i) yeasts with the same name should be very similar to each other; (ii) the name on the label of each strain should be reliable and only one strain should be in each culture; and (iii) the name should be reasonably constant.

Today, there are more than 100 yeast genera and 1,500 species recognized as unicellular ascomycetous or basidiomycetous fungi that produce vegetatively by budding or fission, and that form sexual states, which are not enclosed in fruiting bodies [20,50]. However, these described genera and species only represent a tiny fraction of the yeast biodiversity on this planet. Modelling data from the 1990s suggest that there could be as much as 62,000 undescribed yeast genera comprising 669,000 species yet to be discovered [51]. If these calculations still hold true today, there is still much to do for yeast taxonomists in terms discovering new kinds of yeasts, describing their characteristics and devising effective means for their identification and convenient groupings into genera, species and strains. Nature’s untapped treasure trove of yeasts has much to offer to modern-day experimental yeast research and applications in the fermented food and beverage industries, the biofuel industry, the medical field, and in the biotechnology sector [40].

## 4. The Rising Power of Yeast Genetics 

The first step toward tapping into the genetic diversity of yeasts in Nature’s treasure trove was taken by Øjvind Winge from Carlsberg Laboratory—the same research laboratory in Copenhagen where Hansen isolated the first pure culture of brewer’s yeast, initially named as *Saccharomyces carlsbergensis*. In 1935, this founding Father of Yeast Genetics unequivocally demonstrated the alternation of haplophase and diplophase in four strains of *S. cerevisiae* [15]. His findings were consistent with some of Jan Šatava’s earlier observations in Prague. Four years later, Winge and his colleague Otto Laustsen also confirmed an earlier observation by Guilliermond that *Saccharomycoides ludwigii*—a lemon-shaped yeast with bipolar budding—was heterothallic [15].

Using cells grown from a single-cell isolate of a commercial yeast, Winge and Laustsen went on to develop the first form of tetrad analysis with which they demonstrated Mendelian segregation of genetic traits [52]. These two Danish pioneers were able to keep four isolated ascospores from each ascus apart, and then germinate these meiotic products singly to generate haploid cells. These haploids reproduced vegetatively through budding and mitosis. It is remarkable that while these determined researchers dissected the yeast cells in Copenhagen to understand the basic lifecycle of heterothallic and homothallic yeasts, and Mendelian inheritance of genetic traits important in fermentation, there was a World War raging nearby.

The Danes were not the only ones interested in the genetics of yeast during this time of worldwide hardship. On the other side of the globe, an American couple, was equally busy unearthing the fundamentals of the genetic lifecycle of *S. cerevisiae* and the breeding of new strains. In 1943, in St. Louis, Missouri, Carl and Gertrude Lindegren were first to report the existence of two opposite mating-types in *S. cerevisiae* [53], a and α, and published the first genetic maps of *S. cerevisiae* in 1949 and 1951 [54]. During the same time, Winge and his colleagues unravelled the genetics of sugar utilisation in *Saccharomyces* yeasts [55]. Winge’s work also inspired Urs Leupold to develop the genetics of *S. pombe* in Switzerland [43]. 

Winge obtained his *Saccharomyces* strains from Hansen while Lindgren worked primarily with a strain (EM93) that Emil Mrak isolated from rotten figs in Merced, California [56]. All of Winge’s *Saccharomyces* yeasts are currently named *S. bayanus*, *S. cerevisiae*, *S. paradoxus*, or *S. pastorianus*, the latter being synonymous with the original name of *S. carlsbergensis*. By using yeasts originating from natural and/or industrial settings, the laboratories of Winge and Lindegren interbred their *Saccharomyces* isolates to produce laboratory strains which could be mated to generate diploid cells capable of sporulating to produce asci containing four viable spores. It is therefore important to note that the widely-used modern lab haploid S288C (85% related to EM93) and its ‘BY’ derivatives are strains specifically adapted for experimentation under laboratory conditions but are not robust enough for industrial purposes.

**Figure 2:**
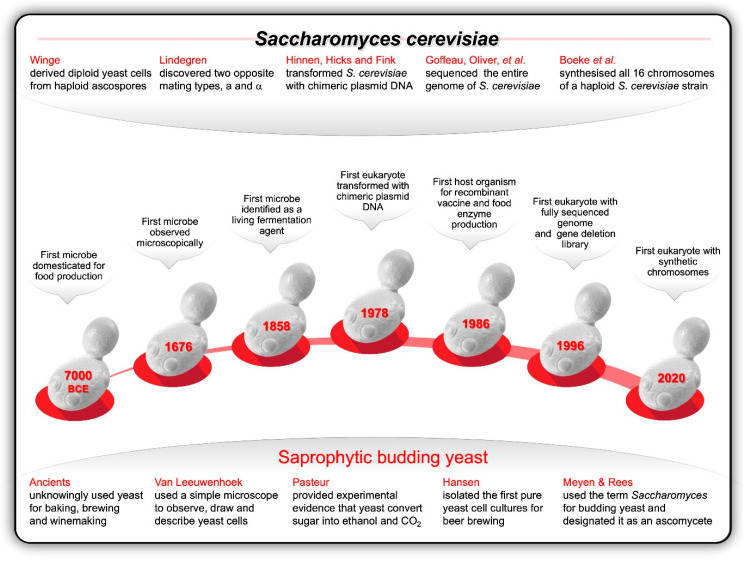
The budding yeast, *Saccharomyces cerevisiae*, has emerged as the most popular eukaryotic model organism to develop and test new genetic technologies and industrial applications. These attributes include its relatively short reproduction time; simple and inexpensive cultivation as stable haploid, diploid and polyploid cells in defined media; efficiency of sporulation and cross-hybridisation between two stable opposite mating-types; ease of mutant isolation and mapping; efficacy of genetic transformation, maintenance of multiple copies of circular plasmids as well as chromosomal integration through homologous recombination; rare pathogenicity; relatively small genome size; and availability of gene deletion libraries consisting of strains carrying unique DNA barcodes that mark each deletion [19,57].

Thanks to the seminal work conducted during the first half of the twentieth century, and the follow-up research conducted by numerous eminent yeast biologists since, the genetics, cell cycle, and life cycle of *S. cerevisiae* are well understood today and has positioned this food-grade yeast as the premier eukaryotic model organism for both genetic and genomic research (Figure 2). *S. cerevisiae* has a compartmentalised sub-cellular structure with an encapsulated nucleus, typical of a eukaryote (*eu* = true; *karyon* = kernel). It is a safe unicellular organism and can be cultured in an uncomplicated, inexpensive way in the laboratory. Under optimal culturing and nutritional conditions, all three basic cell types (a, α, and a/ α) can double their mass every 90 min through an asexual mitotic budding process.

What we know today can be summarised as follows [58]: Heterothallic *S. cerevisiae* a/ α diploids lacking the *HO* mating-type switch gene can be induced to undergo meiosis and sporulate, thereby generating stable haploids of both mating-types (*MAT*a and *MAT* α). In turn, *MAT*a and *MAT* α haploids can mate and give rise to *MAT*a/*MAT* α diploids, capable of sporulating and generating four new ascospores per ascus (tetrad), two of each mating-type. Homothallic haploids can switch their mating-types from *MAT*a to *MAT* α and vice versa, and then self-mate. So, haploid ascospores derived from homothallic diploids can establish diploid lines (i) by mating with their own mitotic daughter cells after a mating-type switching event (haploselfing); (ii) by mating with another sibling ascospore stemming from the same meiotic event (intra-tetrad mating); or, more rarely, (iii) by mating with an unrelated individual (outcrossing) [59]. In homothallic haploids, the *HO* gene can use the information from the silent *HML* and *HMR* loci—located on either side of the *MAT* locus on Chromosome 3—and dictate the switching between *MAT*a and *MA*T α. The haplontic phase of homothallic strains are therefore much shorter than the diplontic phase of their sexual life cycle. Both heterothallic and homothallic strains can asexually reproduce in the *MAT*a and *MAT* α haploid state or state of higher ploidy (from diploids to heptaploids) and aneuploidy (abnormal number of chromosomes per cell, i.e., not 16, 32, 48, etc). Most laboratory strains of *S. cerevisiae* are heterothallic haploid or diploid strains, whereas industrial strains can be either heterothallic or homothallic, and they are diploid, aneuploid, and polyploid. The ability to control the life cycle of *S. cerevisiae* and to switch it between mitotic and meiotic reproduction, and to develop strains with different ploidies surpass the experimental flexibility of any other model organism.

## 5. The Unstoppable Blizzard of Inventive DNA Science

It was unbeknown to Johann Fredrich Miescher in 1869 that his discovery of a mysterious substance (nuclein) in the nuclei of human white blood cells would spark the discovery of the molecular basis of all life. Twenty years later, his student, Richard Altmann, replaced the term nuclein with nucleic acid [60], paving the way for Albrecht Kossel to isolate the five nitrogenous compounds that are present in nucleic acids (adenine, cytosine, guanine, thymine, and uracil) by 1901 [61]. In 1928, Frederick Griffith postulated that this nucleic acid, which he called transformation principle, might be the molecule of inheritance [62]. A year later, Phoebe Levene discovered deoxyribose and showed that the components were linked together in the order phosphate-sugar-base to form units.

By pursuing Griffith’s transformation principle in mutants of the mould *Neurospora crassa*, George Beadle and Edward Tatum conceived the idea, in 1941, that genes act through the production of enzymes, with each gene responsible for producing a single enzyme that, in turn, affects a single step in a metabolic pathway [63]. Subsequently their concept became known as the one-gene-one-enzyme hypothesis. At the time, this hypothesis challenged the widely held belief that it was proteins that served the function of carrying genetic information. However, in 1944, Oswald Avery, Colin MacLeod, and Maclyn McCarty used virulent and avirulent strains of the bacterium *Streptococcus pneumoniae* to demonstrate experimentally that the macromolecule described as the transformation principle by Griffith and defined as a nucleic acid by Levene, was the substance that causes genetic transformation and recombination [64]. In 1946, Joshua Lederberg and Edward Tatum showed that the bacterium *Escherichia coli* can enter a sexual phase during which mating cells exchange (and recombine) genetic information through a process called bacterial conjugation (or transformation) [65]. In 1952, Lederberg and Norton Zinder discovered another genetic recombination process that does not require mating (conjugation) and does not require physical contact between the bacterial cell donating the genetic material and the cell receiving that material [66]. They were testing for recombination in the bacterium *Salmonella typhimurium* when they discovered the horizontal transfer of genetic information from a donor to a recipient cell via a virus particle. Alfred Hershey and Martha Chase showed that when bacteriophages, which are composed of DNA and protein, infect bacteria, their DNA enters the host bacterial cell, but most of their protein does not [67]. This process—known as transduction—was shown to happen through either the lytic cycle of the lysogenic cycle of bacteria when bacteriophages harness the protein synthesis machinery of an infected host cell to produce new viral particles. The discovery of these natural processes of genetic recombination (exchange and reshuffling of genetic material during mating/conjugation, transformation, and transduction) served as the final proof that deoxyribonucleic acid (DNA) was the molecule of heritance and not proteins.

**Figure 3:**
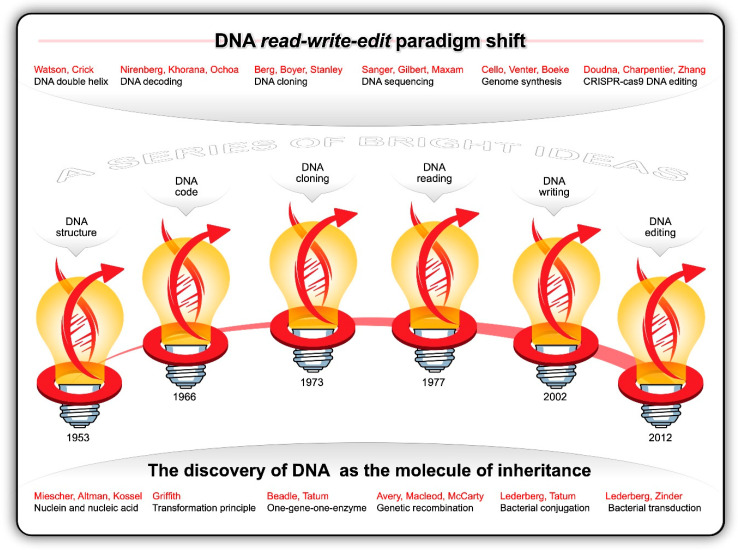
The discovery of DNA is one of our greatest scientific achievements. The history of DNA science consists of a series of bright ideas and profound breakthroughs that led to the current read-write-edit paradigm shift.

Guided by the principles of the scientific method and consilience, researchers from multiple branches of science were stacking micro-insight atop micro-insight—gradually converging on cracking the code of life. Like the innumerable snowflakes that accumulate in thick layers of ice crystals until the weight suddenly shifts and causes an avalanche, each observation and datapoint culminated in the discovery of the structure and function of DNA. Informed by the work and X-ray photographs of DNA fibres generated by Maurice Wilkins and Rosalind Franklin [68], James Watson and Francis Crick postulated, in 1953, the double helix structure of DNA [69,70]. The two complementary polymeric chains of nucleotides twisted about each other in the form of a regular right-handed double helix arguably became the most recognisable symbol of innovative science. Each of the four nucleotides contains a deoxyribose residue, a phosphate group and a purine [adenine (A) and guanine (G)] or pyrimidine [thymine (T) and cytosine (C)] base. The discovery that sequences of A-T and G-C base pairs in the double helix contains the genetic instructions for all living things was nothing short of monumental. This breakthrough catalysed a blizzard of discoveries (Figure 3).

In 1958, Matthew Meselson and Franklin Stahl [71] proved Watson and Crick’s hypothesis correct that, when the double stranded DNA helix is replicated, each of the two new double-stranded DNA helices consisted of one strand from the original helix and one newly synthesized. In the same year Arthur Kornberg and his colleagues isolated DNA polymerase I from *E. coli* and demonstrated that DNA can be replicated in a test tube. In 1960, RNA polymerase was discovered as the enzyme that synthesises ribonucleic acid (RNA) on the surface of single-stranded DNA. The existence of messenger RNA (mRNA) was first hinted at by Jacques Monod and François Jacob, and was subsequently discovered by Jacob, Sydney Brenner, and Matthew Meselson in 1961 [72]. This led to the deciphering of the complete genetic code by Marshall Nirenberg, Gobind Khorana, and Severo Ochoa in 1966 [73]. In essence, the sequence of 3-nucleotide DNA codons of genes is transcribed into nucleotide triplets in mRNA, which, in turn, is translated on ribosomes to proteins. Knowing how the sequence of 61 DNA sense codons (4^3^ minus 3 stop codons) specifies the sequence of the 20 amino acids in proteins through transcription and translation in all living cells and organisms enabled scientists to alter and control the expression of genes and chromosomes.

With the discovery of DNA ligase by the laboratories of Martin Gellert, Robert Lehman, Charles Richardson and Jerard Hurwitz in 1967 and the isolation of the first site-specific restriction enzyme by Hamilton Smith in 1970, it became possible to cut DNA at specific sites and join DNA fragments together. This was the dawn of the recombinant DNA (rDNA) era. In 1971, Paul Berg pioneered gene splicing by creating recombinant DNA from more than one species [74]. In 1973, Herbert Boyer and Stanley Cohen accomplished the first successful genetic engineering experiment by transferring plasmid-borne DNA from one species into another. They created chimeric plasmids, transformed them into *E. coli*, and functionally expressed the cloned genes in the host cells. It was now possible to clone any foreign gene and express them in bacterial host cells.

This breakthrough immediately raised the first scientific concern that cloning and rDNA procedures might generate unsafe novel microorganisms [75]. These scientific concerns preceded what would become more widespread public concerns as the 20th century progressed. These public concerns often occurred with a context of government, scientific, media and public discourse that fed off and often amplified ongoing public debate about the consequences of engineering biology. For example, in 1974 there was a call for a worldwide moratorium on certain rDNA experiments. In the following year, a British Government report called for special laboratory precautions for rDNA research and, at an international meeting in California, attendees urged that researchers adopt guidelines for regulating rDNA experimentation and called for the development of safe bacteria and plasmids that cannot escape the laboratory. In 1976, the National Institutes of Health (NIH) released the first guidelines, restricting several categories of rDNA experimentation. However, there was rising public concern that the NIH guidelines might not be effective. A New York Times Magazine advised against the awarding of the Nobel Prize for rDNA research [76]. This did not deter the creation of the first rDNA molecules containing mammalian DNA and the discovery of introns in split genes of higher eukaryotes. This led to the formation of the first genetic engineering company, Genentech, specifically founded in 1977 to use rDNA to produce medically-important drugs. Somatostatin became the first human hormone to be produced by genetic engineering [77]. There was, however, notable commentary throughout this time that policymakers and the public were being excluded from rDNA policy discussions. The demands of democratic decision making led to the inclusion of wider publics in later discussions of engineering biology policy [78].

Frederick Sanger [79] developed the first polymerization-based DNA sequencing method with which he determined the complete genome sequence of bacteriophage φX174 in 1977. Walter Gilbert and Allan Maxam developed a chemical-modification-based DNA sequencing method [80]. These advances spawned a whole new era of ‘next-generation’ sequencing and the decoding of genes and genomes of hundreds of viruses and prokaryotic and eukaryotic organisms.

Most of the early DNA cloning and sequencing experiments were conducted with bacteria and bacteriophages. This changed in 1978 when Albert Hinnen, James Hicks, and Gerald Fink developed a transformation procedure for *S. cerevisiae* [81]. For the first time researchers were able to transfer plasmids carrying cloned DNA into a eukaryotic cell. This fundamental step-change in molecular synthetic gene array yeast genetics catalyzed the development of a plethora of molecular tools, such as libraries of barcoded gene deletion and overexpression strains, and the use of synthetic gene arrays as a high-throughput technique for exploring synthetic lethal and synthetic sick genetic interactions [82]. Thereafter, this yeast became an ideal food-grade eukaryotic model organism for genetic engineering studies and applications in the ‘biotech’ industries. For example, during the 1980s, *S. cerevisiae* was used to produce the first genetically modified (GM) vaccine (against hepatitis B) and the first GM food enzyme (the milk coagulation enzyme, chymosin, for cheese-making). In 1996, *S. cerevisiae* became the first eukaryote whose genome [~12 Mb (non-redundant) to ~14 Mb (total) genome carrying ~ 6000 genes on 16 chromosomes varying in length from ~200 to ~2000 kb] was fully sequenced [83,84]. 

Work on *S. cerevisiae* pioneered several of the major breakthroughs during the latter part of the previous century, which concluded with Dolly, the first cloned sheep, and the announcement of the first draft of the human genome sequence. By the start of the new millennium, it was possible to clone any gene into most organisms and read the DNA code of any genome in a short period of time. This sparked genuine concerns in some parts of society that technological innovation was outpacing the ability of laws and regulations to keep up [85]. As we entered the new millennium, a few paradigm shifts were on the cards that would increase authorities’ concerns about technological governance.

## 6. The Paradigm Shift from DNA Reading to DNA Writing and DNA Editing

Parallel to the 20th century seismic shift in genetic modification and DNA ‘reading’, there was another seismic shift occurring in the information and computing sciences. With the dawn of the 21st century, these two transformational forces began to converge. Rapid advances in DNA sequencing and DNA synthesis would be translated to the abstraction, characterisation and cataloguing of life’s design and solution space [57,86]. This DNA read-write-edit revolution in biology enabled the construction of new biological parts (genes), devices (gene networks) and modules (biosynthetic pathways). As biological functionality entered higher levels of abstraction a phase change occurred in the scale and scope of applied biological system design (cells and organisms) [87,88].

This shift in capacity saw the design-build-test-learn process adopted by the emerging field of synthetic biology. Engineering processes were adopted in order to rationalise biological device design [89]. The chemical synthesis of artemisinin in *S. cerevisiae* [90] was the first major achievement in biodesign. The decade-long timeframe for this project quickly began to seem archaic. Gene editing methodologies like zinc finger endonucleases [91] and TAL effector nucleases (TALEN) [92] mediated sequence-specific recognition of DNA soon gave way to the sequence-specific binding capabilities of CRISPR-Cas [93]. With a broad host range, CRISPR vastly widened the design space of biological system engineering.

**Figure 4:**
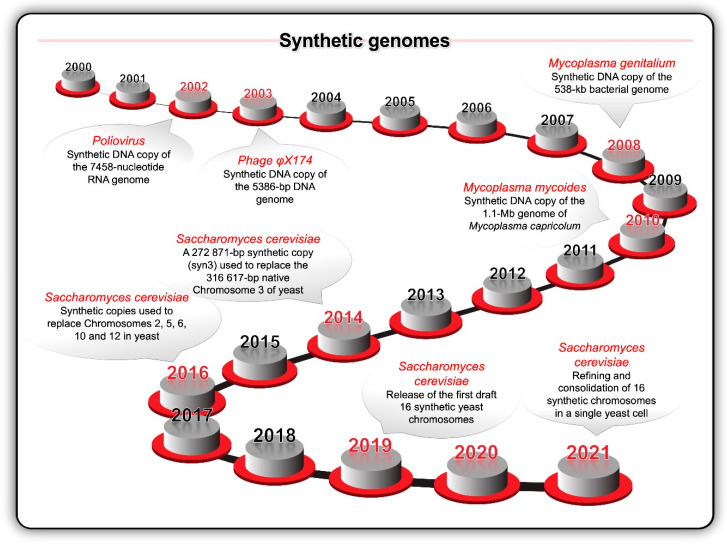
Key milestones in terms of the synthesis of viral and bacterial genomes inspired the idea to chemically synthesise the 16 chromosomes of the yeast *Saccharomyces cerevisiae* and replace the native chromosomes with the synthetic chromosomes.

Key milestones (Figure 4) during the opening decades of the 21st century included the synthesis of the first viral (poliovirus in 2002 and bacteriophage φX174 in 2003) and bacterial (*Mycoplasma genitalium* in 2008 and *Mycoplasma mycoides* in 2010) genomes [94,95,96,97]. *S. cerevisiae* became the target of a large international project to synthesise all 16 chromosomes—the Synthetic Yeast Genome (Yeast 2.0 or Sc2.0) project [41]. Meanwhile, similar advances were occurring across the full suite of enabling computer assisted design (CAD) tools and publicly accessible genomic databases. In 2010, the University of California Berkeley hosted the inaugural Critical Assessment of Genome Interpretation (CAGI) competition [86]. Modern biodesign CAD makes use of software as a service (SaaS) methodology like browser-based applications, cloud computing, and machine learning.

**Figure 5:**
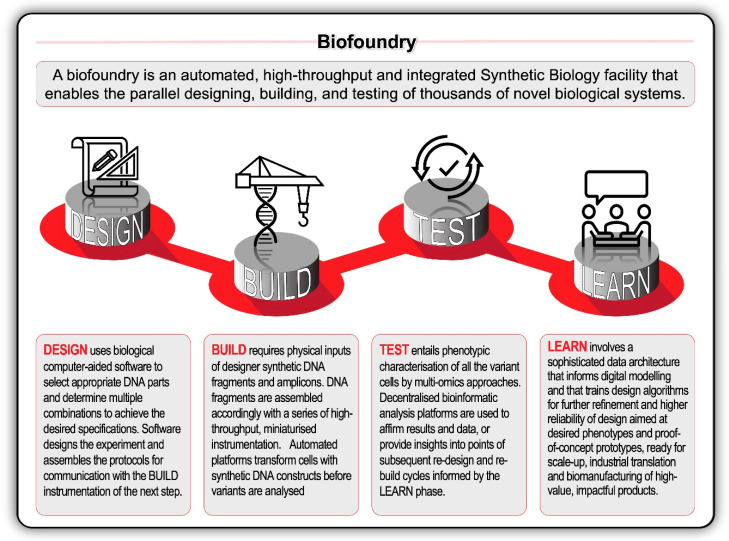
The application of design-build-test-learn (DBTL) biological engineering cycle can be accelerated in high-throughput, automated biofoundries with robotic workflows and technology synthetic biology platforms. Recent rapid advances in high-throughput DNA sequencing (reading) and DNA synthesis (writing and editing) techniques are enabling the design and construction of new biological parts (genes), devices (gene networks), and modules (biosynthetic pathways), and the redesign of biological systems (cells and organisms) for useful purposes.

The biodesign transformation was encapsulated by the growth of the International Genetically Engineered Machine (iGEM) competition from its inauspicious 2003 beginnings. From an independent study course at the Massachusetts Institute of Technology (MIT), iGEM has grown to host over 40 000 students and a database with more than 20 000 standard biological parts. This scale is matched by the upstream and downstream companies servicing the design-build-test-learn ‘full stack’ of biological engineering. Biological design and bioinformatic software solutions are now provided by companies like Benchling, Synthrace, Ryffin, and Teselagen. Other companies—like Ginkgo Bioworks, BGI and Twist Bioscience—form vertically integrated biodesign ‘stacks’ or horizontal ‘platforms’ [89]. Genome foundries, or biofoundries, have further revolutionised biodesign through the deployment of high-throughput technologies combined with automated and modular workflows (Figure 5).

The biofoundry revolution is encapsulated by commercial technologies like Antheia, and the 2019 announcement of the Global Biofoundries Alliance [98]. Together, the research and commercial biofoundry sectors are enabling one of this century’s largest grand challenge projects—the Genome Project Write. Similar to how the 20th century’s Human Genome Project followed on the heels of the sequencing of S. cerevisiae, so too will the Genome Project Write follow in the footsteps of Sc2.0. In November 2019, at the Genome Project Write and 8th Annual Sc2.0 meeting in New York, the international Yeast 2.0 Consortium announced the first draft of the 16 synthetic chromosomes of *S. cerevisiae.* Although there remains work to be undertaken to ensure that the growth of a strain carrying these redesigned chromosomes in a single cell would be on par with that of the original strain, one can safely assume that *S. cerevisiae* is about to become the first eukaryote to be powered by a chemically-synthesised genome.

Importantly, within the same timeframe it has taken the Sc2.0 project to synthesise *S. cerevisiae*, questions of dual-use research of concern have become much more interwoven with developments in the basic and applied research of synthetic biology. The National Academies of Science Engineering and Medicine began to look more critically at developments in synthetic, systems, and engineering biology [99,100,101]. Disciplines in the humanities began to explore the wider impacts of synthetic biology on foreign policy, international relations and international economics [102]. The tools and techniques arising from the genetic read-write-edit revolution were beginning to have profound ethical, legal, and social implications (ELSI). The practice of science communication had never been more important in ensuring the work of technicians, scientists, and entrepreneurs continued to be in alignment with public values and public interests. This did not go unnoticed by the maturing synthetic biology community, and just as the Human Genome Project dedicated significant funds to ELSI research, the Genome Project Write has made the same commitment [103].

## 7. Back to the Future of Biodesign

The reimagining of life as information has fundamentally altered the design space of biology. While the first two decades of the 21st century were characterised by revolutions in chemical synthesis and biodesign, the next two decades will be characterised by a revolution in multiple length scale (multiscale) engineering. Engineering biology is about to move beyond biomimicry and materials design to the rational design of information communication between biological and digital systems. Emerging areas like optogenetics, bioelectrochemistry, and quantum biology are important fields to watch. Importantly, developments in quantum computing are going to have implications for the systems modelling of biological function at the quantum level. When one considers the potential of combining quantum computing, systems biology, and machine learning led in silico biodesign, the true scale of the biological design space available for engineering is difficult to imagine.

At the same time, there is a growing trend of challenge-focused research in the field of engineering biology. The 2019 research roadmap released by the Engineering Biology Research Consortium (EBRC) highlighted five application and impact sectors [104]. This is coupled with a growing focus on the bioeconomy and ensuring a globally sustainable future through circular economy models. In agriculture, this change is visible in attempts to commercialise milk synthesis, wheat synthesis, and protein synthesis. Then there is the focus on synthesising food ingredients, such as resveratrol, vanillin, stevia, nootkatone, and saffron.

Just as information technologies were demarcated a general-purpose science and technology in the 20th century, so too is it becoming clear that the disruptive potential of engineering biological systems will impact on every economic sector in the mid-21st century. This mega-trend is born from the consilience of the sciences, but also the consilience of challenge-focused research and the multidisciplinarity of STEMM-HASS collaboration. This is a process that dates back to the creation of the scientific method and the creation of the concept of consilience [22]. Since that time, the world has been fundamentally transformed. At the heart of this transformative journey is yeast, an organism that has symbiotically evolved with human civilization for over 8,000 years. Whether it be space travel, the reuse of gaseous carbon waste, food 3.0, or next-generation vaccine manufacturing platforms—yeast continues to offer a biological solution to humanity’s grand challenges. However, unlike information technologies, biological systems can be engineered through harnessing the natural processes of evolution. In many ways, this makes them technologically superior to the inorganic substrates inherent to computing. Machine learning approaches may accelerate in silico biodesign, but only biological devices can synthesise chemicals while simultaneously operating as biosensory arrays. The true potential of Von Neumann’s universal constructor is clear. The 21st century will be built on the only technology that can recreate itself—life.
